# Supplementary material for: Pleiotrophin and the Expression of Its Receptors during Development of the Human Cerebellar Cortex
Source: Cells. 2023 Jun 27;12(13):1733. doi: 10.3390/cells12131733 (PMC10341181; doi:10.3390/cells12131733)
Supplement: Supplementary file 1 [file cells-12-01733-s001.zip › cells-2410250-supplementary.pdf]

Supplementary Material

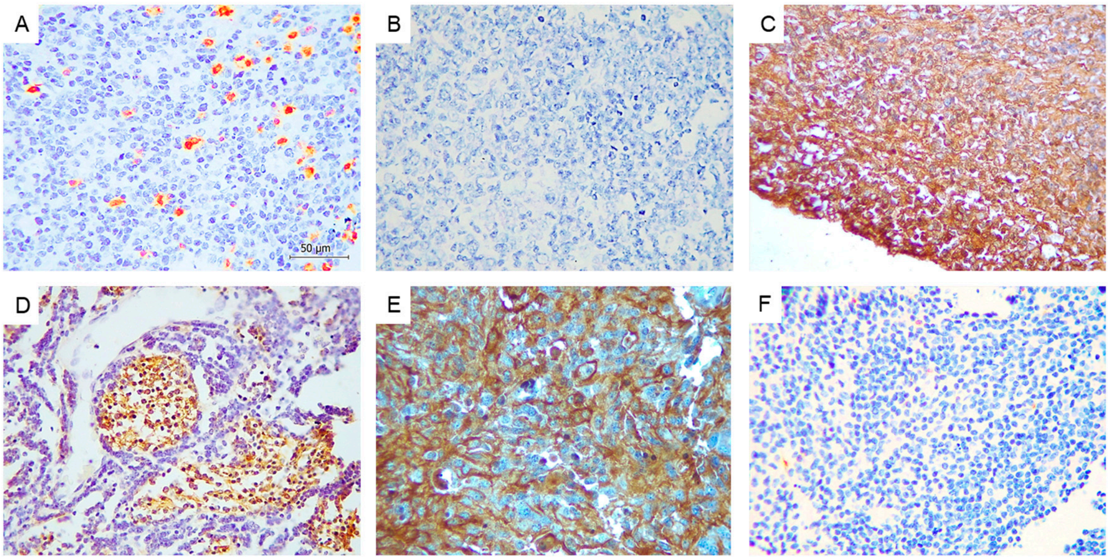

Figure S1. PTPRZ1 control tissue. **(A)** Lymphoid follicles of cecal appendix tissue with positive stain reaction in germinal center cell cytoplasm. PTPRZ1 expression has been demonstrated in human mature B cells, with important participation in the regulation of cell survival mechanisms [61]. **(B)** Negative control in appendix cecal tissue (without primary antibody). **(C)** Immature brain cortex tissue with cytoplasmic PTPRZ1 diffuse stain reaction, with moderate to intense staining in neurons, cytoplasm, and neuropil. PTPRZ1 expression has been demonstrated in neurons and astrocytes of the developing cerebral cortex and hippocampus in mammals, as well as in human neuroblastoma cells [62,63]. **(D)** Desmoplastic/nodular medulloblastoma tissue shows a positive cytoplasmic stain reaction into the nodules. **(E)** Classic medulloblastoma shows positive stain reaction in neoplastic cell cytoplasm. **(F)** Classic medulloblastoma shows negative reaction to PTPRZ1. Although RPTPZ1 expression has been demonstrated in neuroblastoma cells, there are no records of its expression in medulloblastoma cells. In our group, we have identified differential expression in subgroups of this type of neoplasm (unpublished data).

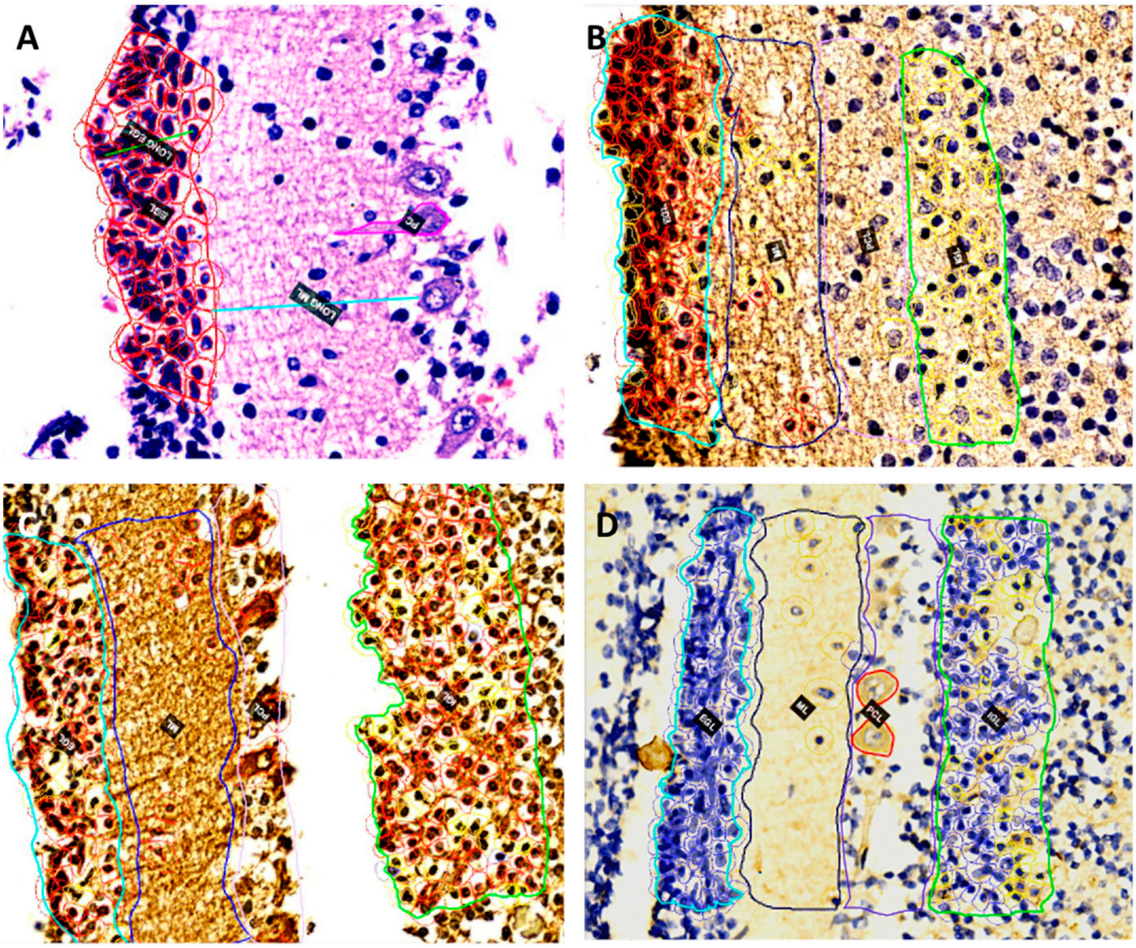

Figure S2. Histological and immunohistochemistry analysis. **(A)** The red circles indicate the limited cytoplasm of granule cells in EGL; the green line shows the thickness of EGL; the blue line specifies the thickness of ML. The pink structure marks the PC somas. **(B–D)** The red circle indicates cells with strong intensity (3+); the yellow circle shows cells with moderate intensity (2+); the orange circle limits the cell with weak intensity (1+); and the blue circle shows the negative cell. The big rectangle separates each cerebellar cortex layer [38].

REFERENCES OF THE SUPPLEMENTARY MATERIAL

61. Cohen, S.; Shoshana, O.; Zelman-Toister, E.; Maharshak, N.; Binsky-Ehrenreich, I.; Gordin, M.; Hazan-Halevy, I.; Herishanu, Y.; Shvidel, L.; Haran, M.; et al. The Cytokine Midkine and Its Receptor RPTPζ Regulate B Cell Survival in a Pathway Induced by CD74. *J. Immunol.* **2012**, *188*, 259–269, doi:10.4049/jimmunol.1101468.

62. Shintani, T.; Watanabe, E.; Maeda, N.; Noda, M. Neurons as Well as Astrocytes Express Proteoglycan-Type Protein Tyrosine Phosphatase ζ/RPTPβ: Analysis of Mice in Which the PTPζ/RPTPβ Gene Was Replaced with the LacZ Gene. *Neurosci. Lett.* **1998**, *247*, 135–138, doi:10.1016/S0304-3940(98)00295-X.

63. Del Campo, M.; Fernández-Calle, R.; Vicente-Rodríguez, M.; Martín Martínez, S.; Gramage, E.; Zapico, J.M.; Haro, M.; Herradon, G. Role of Receptor Protein Tyrosine Phosphatase β/ζ in Neuron–Microglia Communication in a Cellular Model of Parkinson’s Disease. *Int. J. Mol. Sci.* **2021**, *22*, 6646, doi:10.3390/ijms22136646.

38. Bankhead, P.; Loughrey, M.B.; Fernández, J.A.; Dombrowski, Y.; McArt, D.G.; Dunne, P.D.; McQuaid, S.; Gray, R.T.; Murray, L.J.; Coleman, H.G.; et al. QuPath: Open Source Software for Digital Pathology Image Analysis. *Sci. Rep.* **2017**, *7*, 16878, doi:10.1038/s41598-017-17204-5.
